# An Evaluation of Dose Equivalence between Synchrotron Microbeam Radiation Therapy and Conventional Broadbeam Radiation Using Clonogenic and Cell Impedance Assays

**DOI:** 10.1371/journal.pone.0100547

**Published:** 2014-06-19

**Authors:** Mohammad Johari Ibahim, Jeffrey C. Crosbie, Yuqing Yang, Marina Zaitseva, Andrew W. Stevenson, Peter A. W. Rogers, Premila Paiva

**Affiliations:** 1 Department of Obstetrics and Gynaecology, Royal Women’s Hospital, University of Melbourne, Parkville, Victoria, Australia; 2 Faculty of Medicine, Universiti Teknologi MARA, Sungai Buloh Campus, Jalan Hospital, Sungai Buloh, Selangor, Malaysia; 3 William Buckland Radiotherapy Centre, Alfred Hospital, Melbourne, Australia; 4 Australian Synchrotron Imaging and Medical Beamline, Clayton, Australia; 5 CSIRO Materials Science & Engineering, Clayton, Australia; Mizoram University, India

## Abstract

**Background:**

High-dose synchrotron microbeam radiation therapy (MRT) has shown the potential to deliver improved outcomes over conventional broadbeam (BB) radiation therapy. To implement synchrotron MRT clinically for cancer treatment, it is necessary to undertake dose equivalence studies to identify MRT doses that give similar outcomes to BB treatments.

**Aim:**

To develop an *in vitro* approach to determine biological dose equivalence between MRT and BB using two different cell-based assays.

**Methods:**

The acute response of tumour and normal cell lines (EMT6.5, 4T1.2, NMuMG, EMT6.5ch, 4T1ch5, SaOS-2) to MRT (50–560 Gy) and BB (1.5–10 Gy) irradiation was investigated using clonogenic and real time cell impedance sensing (RT-CIS)/xCELLigence assays. MRT was performed using a lattice of 25 or 50 µm-wide planar, polychromatic kilovoltage X-ray microbeams with 200 µm peak separation. BB irradiations were performed using a Co^60^ teletherapy unit or a synchrotron radiation source. BB doses that would generate biological responses similar to MRT were calculated by data interpolation and verified by clonogenic and RT-CIS assays.

**Results:**

For a given cell line, MRT equivalent BB doses identified by RT-CIS/xCELLigence were similar to those identified by clonogenic assays. Dose equivalence between MRT and BB were verified *in vitro* in two cell lines; EMT6.5ch and SaOS-2 by clonogenic assays and RT-CIS/xCELLigence. We found for example, that BB doses of 3.4±0.1 Gy and 4.40±0.04 Gy were radiobiologically equivalent to a peak, microbeam dose of 112 Gy using clonogenic and RT-CIS assays respectively on EMT6.5ch cells.

**Conclusion:**

Our data provides the first determination of biological dose equivalence between BB and MRT modalities for different cell lines and identifies RT-CIS/xCELLigence assays as a suitable substitute for clonogenic assays. These results will be useful for the safe selection of MRT doses for future veterinary and clinical trials.

## Introduction

Synchrotron microbeam radiation therapy (MRT) is an experimental form of radiation therapy in which synchrotron-generated X-rays are segmented by a collimator, producing intense microbeams, tens of µm wide separated by hundreds of µm. Synchrotron MRT has been used to ablate tumours in animal models at radiation levels that spare normal tissues [1], [2], [3], [4], [5], [6], [7], [8], with an apparent increase in the therapeutic index of up to 5-fold over conventional/broadbeam (BB) radiotherapy [1], [4]. There is no clear explanation as to why MRT gives a higher therapeutic index over BB irradiation.

**Figure 1:**
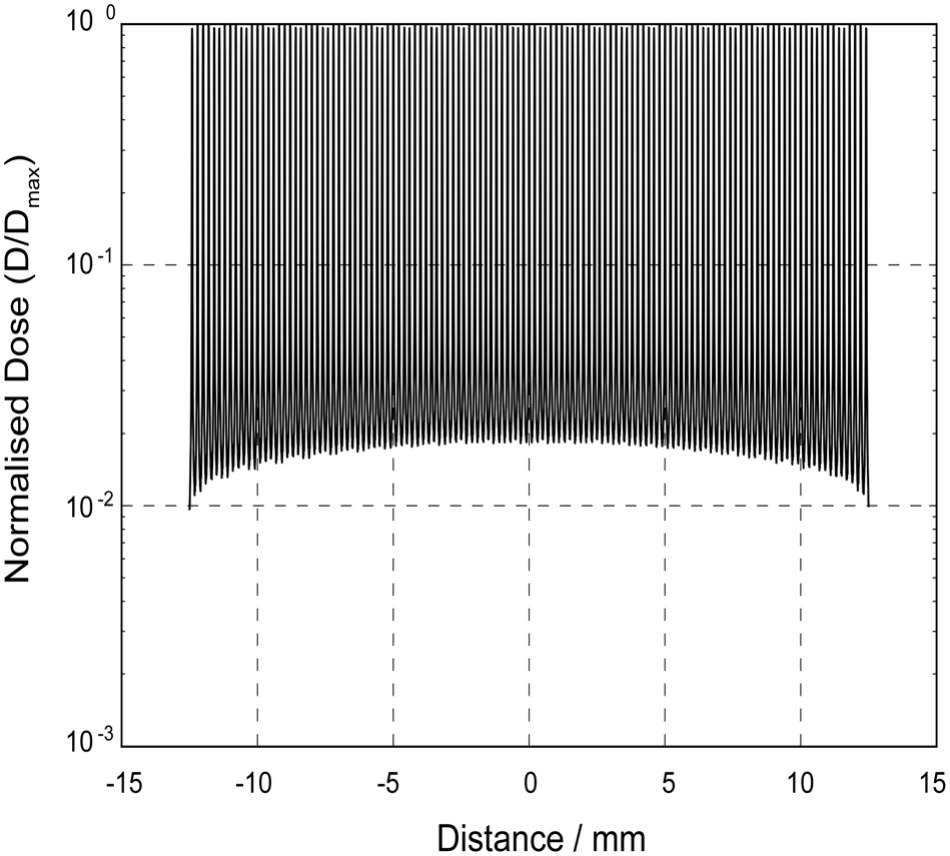
The different dose components of the MRT beam. MRT ‘peak’ & ‘valley’doses, This profile was generated from our GEANT4 Monte Carlo computer simulations of the dose distribution for a 25×25 mm array of 25/175 µm microbeams at a depth of 2 mm in a water phantom. The Supporting Information section contains more detailed information on the simulations.

One of the major hurdles to implementation of MRT as a therapeutic option for cancer is to identify the optimal radiation dose to treat different tumours. In devising an approach to this task, it is important to recognise that MRT is fundamentally different to BB. The physical dosimetry associated with MRT is more complex than that for conventional BB radiotherapy because there are different components to a MRT dose profile [9], [10], [11], [12] (Figure 1).

Typical in-beam or peak radiation doses are 350–1000 Gray (Gy), whereas doses between adjacent microbeams (i.e. valley doses) are of the order 5–20 Gy. MRT utilizes kilovoltage energy X-rays (50–250 keV) rather than the megavoltage energies produced by hospital linear accelerators, in order to maintain spatial fractionation deep into the tissues. In addition to the ‘peak’ and ‘valley’ dose there is also the ‘integrated’ dose which is the microbeam dose averaged over the entire irradiation area [3]. The Peak-to-Valley Dose Ratio (PVDR) is another important physical parameter in MRT dosimetry and is determined by several factors including the collimator geometry (i.e. the microbeam width and the centre-to-centre spacing). Therefore, the effects of MRT on biological tissue are likely dependent on a number physical parameters.

The dosimetry for the MRT studies presented here was based on Monte Carlo computer simulation. The dosimetry for the MRT studies presented here was based on Monte Carlo computer simulation. The Monte Carlo method predicts a physical deposition of energy. The biologically equivalent dose described here is not the same as the physical valley dose but rather is the dose of BB radiation which elicits the same biological effect in cells as MRT. We are not attempting to measure the physical dose. The biological equivalent dose takes into account the different dose components of MRT; peak, valley, integrated dose. This equivalence in dose is the novel aspect of our work; we take an MRT dose distribution and we can state what the equivalent biological response is with a BB dose distribution.

Current clinical radiotherapy protocols have been optimised over many years using empirical approaches. An empirical approach to optimising MRT for clinical use is not acceptable in the modern era; hence the need to undertake dose equivalence studies to identify MRT doses that give similar outcomes to BB treatments.

Clonogenic assays are considered the “gold-standard” for evaluating the biological response (i.e. cytotoxicity) to irradiation. As an alternative to clonogenic assays a study by Roa et al. [13] evaluated real-time cell-impedance sensing (RT-CIS). RT-CIS measures the electrical impedance of cells across gold electrodes in the base of a culture plate. The resulting ‘cell index’ is an integrated measure of not only cell number but also cell metabolism and morphology. Roa *et al.* [13] concluded that the results from RT-CIS agreed with the clonogenic data, providing sensitive, dynamic and quantitative measurements, and most significantly shortening the testing time from 14 days (in clonogenic assays) to only 72 hours.

The benefits of RT-CIS (as a substitute for clonogenic assays) apart from the significant time/cost saving is the ability to conduct high-throughput screening (at a number of radiation doses). This is of particular relevance to studies using synchrotron MRT where facility access is limited and large numbers of experiments are planned to fit in over a few days of beam time availability.

In this study, we developed an *in vitro* approach to determine biological dose equivalence between MRT and BB. We first investigated the acute response of different cell lines to MRT and BB using conventional, clonogenic assays and novel, RT-CIS/xCELLigence cell-based assays. For this part of the study, the BB doses we used were approximately equal to the MRT valley dose. This was based on the assumption that the valley dose was the significant contributor to the *in vitro* biological response. Using these results, we then calculated BB doses that we predicted would generate biological responses similar to that of MRT. Finally, using clonogenic and RT-CIS/xCELLigence assays we tested whether these BB doses were indeed biologically equivalent to MRT.

## Methods

### Cell Culture

Mouse mammary tumour cell lines (EMT6.5, 4T1.2 [8], EMT6.5ch, 4T1ch5) were provided by A/Prof. Robin Anderson from Peter MacCallum Cancer Centre, East Melbourne, Victoria, Australia. The EMT6.5 cell line was derived from the mouse mammary carcinoma cell line EMT6 [14] by single-cell cloning [15]. The 4T1.2 [16] [17] and 4T1ch5 cell lines were derived from the 4T1 cell line [18]. The mCherry (ch) fluorescent protein serves as a useful tag that can be tracked for example using optical imaging techniques.

The normal mouse mammary epithelial cell line NMuMG was purchased from the American Type Culture Collection (ATCC, Manassas, VA, USA). The osteosarcoma cell line, SaOS-2 (ATCC, Manassas, VA, USA) was provided by A/Prof. Damian Myers from St Vincent’s Hospital, Fitzroy, Victoria, Australia. Cells were cultured in Dulbecco’s modified Eagle’s medium (DMEM) consisting of 4.5 g/L mmol D-glucose and 25 mM HEPES (Invitrogen, Life Technologies, Mulgrave, Australia) supplemented with 10% Fetal Bovine Serum, 100 IU mL^−1^ penicillin G, and 100 µg mL^−1^ streptomycin (Gibco, Life technologies, Mulgrave, Australia) in a humidified incubator with 5% CO_2_ at 37°C.

### Synchrotron Source and Irradiations

MRT irradiation was carried out at the Australian Synchrotron’s Imaging and Medical Beamlime (IMBL), Clayton, Victoria in two experimental sessions in May (session I) and June (session II), 2013. The ‘raw’ synchrotron X-ray beam was produced by a superconducting multi-pole wiggler operating at a peak magnetic field of 3 Tesla. The MRT beam was filtered by in vacuo absorbers, notably 21.2 mm of graphite and 2.83 mm Cu, to produce a polychromatic beam with a mean energy of ∼100 keV for the MRT irradiations.The dose rate in air (air kerma rate) of the incident, synchrotron broad beam was measured by a free-air ionisation chamber using the methodology described in Crosbie et al. (2013) [19]. A tungsten-carbide collimator segmented the incident beam into an array of microbeams. The collimators used in this study produced beam widths of 25 µm or 50 µm, with centre-to-centre spacings of 200 µm. The microbeam collimators at the Imaging & Medical Beamline of the Australian Synchrotron were manufactured by UNT in France and their geometry (beam width, separation) was confirmed during X-ray imaging sessions with high resolution detectors at the European Synchrotron Radiation Facility (ESRF) in Grenoble, France prior to the collimators being shipped to Australia. The PVDR for the 175/25 µm and 150/50 µm collimators were estimated using GEANT4 Monte Carlo computer simulations as approximately 90 and 70 respectively for a 25 mm×25 mm array at a depth of 2 mm in a water phantom. More detailed information on the GEANT4 Monte Carlo simulations appears as Material S1.

Cell lines were cultured in T12.5 flasks as a monolayer until 70–80% confluence. For MRT irradiations, flasks were filled with culture medium and positioned vertically on the platform in front of the path of the beam and irradiated in a stepwise fashion from top to bottom and then from left to right using a ‘step-and-shoot’ technique [20]. The average irradiation time per flask was 6–8 min depending on the dose. The total field size required to cover the flask was 50×40 mm.

For BB irradiations, flasks were sealed and cells irradiated using a Co^60^ teletherapy Unit at the Walter Eliza Hall Institute (WEHI, Melbourne, Australia) delivering approximately 2 Gy/min. For synchrotron BB irradiations, the collimator was removed and irradiations carried out as described above for MRT. The incident beam was filtered by 21.2 mm of flexible graphite (density = 1.0 g/cm^3^), 1.41 mm of (in vacuo) Cu and 2.83 mm of Mo. An additional 2 mm of copper was placed *ex vacuo* in the synchrotron beam path in order to reduce the flux rate to within the mechanical tolerance of the shutters and permit relatively low doses of synchrotron BB radiation to be delivered to the cells. The extra copper and molybdenum filtration increased the mean energy of the broad beam to about 140 keV.

BB doses used during our first experimental session (May 2013) at the Australian synchrotron were chosen based on the GEANT4 Monte Carlo-calculated valley dose for each given MRT dose. Assuming a PVDR of ∼75 (ie: slightly lower than the 175/25 µm PVDR and higher than the 150/50 µm PVDR) we compared the following BB doses versus MRT peak doses - 1.5 Gy BB vs. 112 Gy MRT, 3.8 Gy BB vs. 280 Gy MRT, and 7.5 Gy BB vs 560 Gy MRT). In each case, the BB dose was estimated to be approximately equivalent to the MRT valley dose.

BB doses used during our second session at the Australian synchrotron (June 2013) were chosen based on the results from our first session (refer to *clonogenic assays/data interpolation*).

Following irradiation, flasks were incubated for 4 hours at 37°C/5% CO_2_ to standardise the duration for which cells were incubated in each experiment prior to processing. Cells were then trypsinized and cell densities adjusted for clonogenic or RT-CIS/xCELLigence (Roche Applied Biosystems/ACEA Biosciences, San Diego, CA, USA) assays.

### Clonogenic Assays and Data Interpolations

For clonogenic assays, we used the modified clonogenic assay technique of Puck and Marcus [21] and Franken [22]. Briefly, cells were trypsinized and appropriate cell numbers (100 cells-25×10^3^ cells) were seeded in 10 cm culture dishes in 10 ml growth media for 10–14 days. Colonies were fixed and stained with toluidine blue/70% ethanol for 5 min. The plates were scanned using a gel image scanner (Image Scanner III, Labscan 6, GE Healthcare, Rydalmere, New South Wales, Australia) and the colonies counted using Metamorph software v7 (Molecular Device, LLC, Sunnyvale, CA, USA). Each assay was performed in triplicate and only colonies containing at least 50 cells were counted. The number of surviving colonies (irradiated cells) were normalised against the number of colonies in control plates (non-irradiated cells).

Using data modelling we calculated BB doses that would be biologically equivalent to a given MRT dose. Briefly, survival curves (a semi-log plot of survival versus dose) were generated (from data derived from our first experimental session) and using GraphPad Prism v5 software, data were fitted to the commonly used linear-quadratic model (using least squares minimization) that describes the relationship between cell survival and irradiation dose: 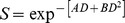 the constants ‘A’ and ‘B’ are cell specific radiosensitivity parameters and describe the survival (S) with increasing dose (D) [23]. (The alpha and beta constants are now included in Table S1 along with the uncertainty (standard error). Using GraphPad Prism v5 software and this model, we were able interpolated the BB doses that would generate equivalent clonogenic survival to that of MRT.

### Real-time Cell Impedance Sensing xCELLigence Assays

We evaluated real-time cell impedance sensing for assaying cell response to radiation using the xCELLigence system (Roche). The impedance measurement provides quantitative data on the biological status of the cells, with the resulting ‘cell index’ reflecting an integrated measure of cell number, metabolism and morphology. Cells were seeded at 2–5×10^3^ cells/per well in triplicate in 96-well E-plates. Cell attachment and growth were monitored and impedance measurements recorded every hour for 96 h–120 h. Data analysis were performed using RTCA Software 1.1 by two methods i) the ‘Slope’ function to calculate the rate at which cell index changed over time (i.e. for graphical purposes) or ii) the ‘DRC’ function to generate sigmoidal dose response curves; 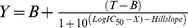 where B and T represent the Bottom and the Top of the sigmoidal curve and the IC_50_ represents the dose required to reduce the cell index measurement by 50% (The alpha and beta constants are now included in Table S1 along with the uncertainty (standard error). BB doses that would generate a similar cell index output to MRT were interpolated. All data were normalised against controls for each experiment.

### Statistical Analysis

All data are expressed as mean ± SEM. All data interpolations were performed using GraphPad Prism version 6.00 for Windows (GraphPad Software, San Diego, California, USA, www.graphpad.com). Statistical analysis was conducted by an independent Student’s t-test or Mann Whitney test (p<0.05 considered significant) using SPSS software version 20 (SPSS Inc, Chicago, IL, USA).

## Results

### Experimental Session (I) at the Australian Synchrotron

#### Evaluation of BB versus MRT using clonogenic assays

**Figure 2:**
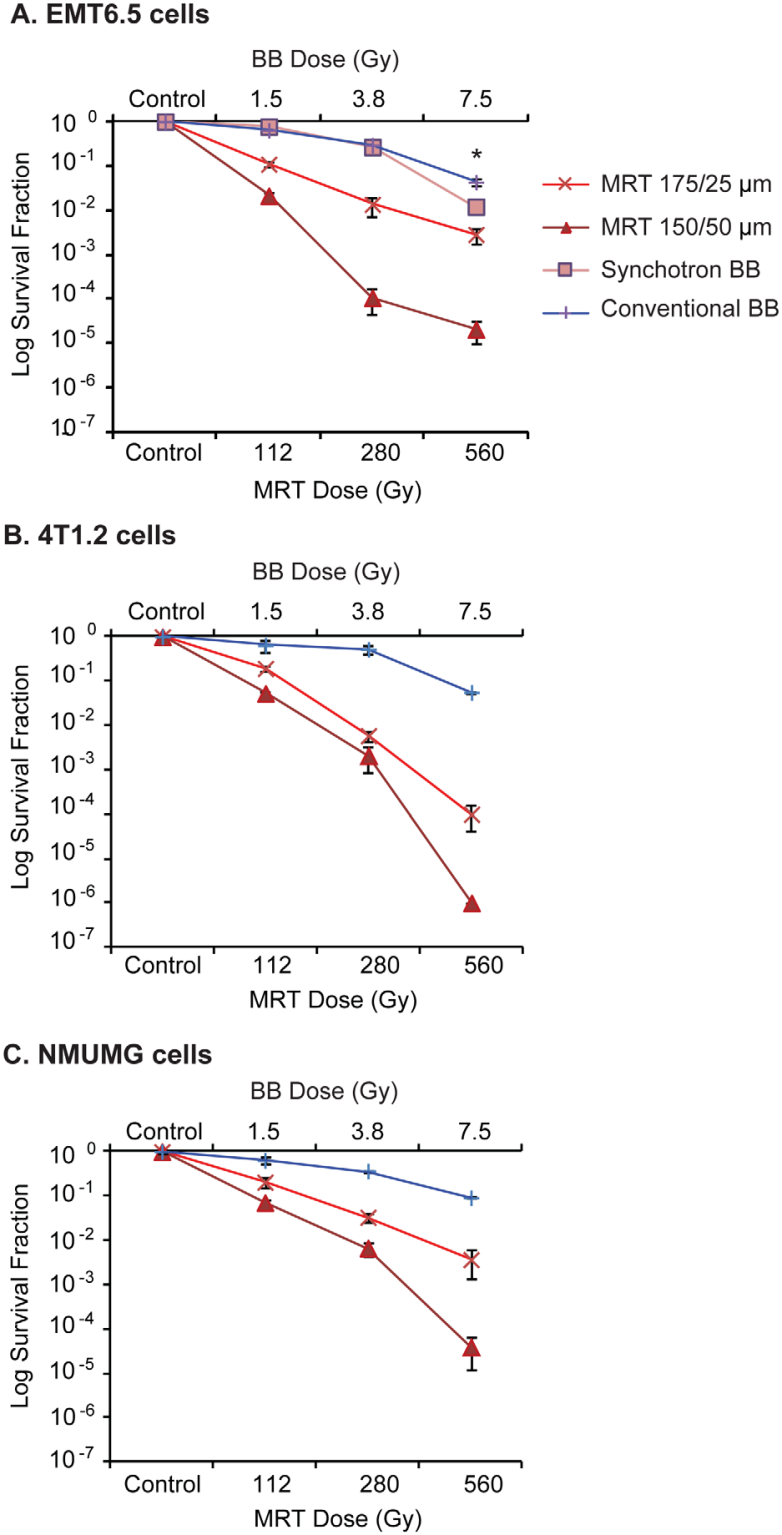
Clonogenic survival of EMT6.5, 4T1.2 and NMuMG cells following conventional/synchrotron broadbeam and MRT irradiations. (A) EMT6.5 tumour, (B) 4T1.2 tumour and (C) NMuMG normal mouse mammary epithelial cells. BB doses were chosen to match the MRT valley dose, based on Monte Carlo computer simulations. Clonogenic survival for 150/50 µm<175/25 µm<BB. 7.5 Gy Synchrotron BB significantly reduced clonogenic survival compared to 7.5 Gy conventional BB (p<0.0001). Data are represented as mean ± SEM (n = 3).

Clonogenic survival at each dose point was lower for 175/25 µm MRT than BB (BB dose≤MRT valley dose) for both the mouse tumour cell lines EMT6.5 and 4T1.2 (Figure 2 A and B) and the normal mouse epithelial cell line NMuMG (Figure 2 C). Further, the 150/50 µm collimator reduced clonogenic survival compared to the 175/25 µm collimator, with marked differences demonstrated in EMT6.5 cells at 280 Gy and 560 Gy (Figure 2 A) and in 4T1.2 and NMuMG at 560 Gy (Figure 2 B and C). All results obtained for BB and MRT were reproducible (n = 3).

#### Evaluation of synchrotron BB versus conventional BB using clonogenic assays

There were no differences in clonogenic survival of EMT6.5 cells when irradiated with synchrotron BB versus conventional BB at 1.5 Gy and 3.8 Gy (Figure 2 A). However, 7.5 Gy Synchrotron BB significantly reduced clonogenic survival compared to 7.5 Gy conventional BB (p<0.0001).

#### Evaluation of BB versus MRT using xCELLigence assays

The cell index (during the log phase of the cell cycle) changed in a dose dependent manner over time (Figure S1). These changes in cell index were dependent on the number of cells seeded in the xCELLigence E-plates. At higher cell seeding densities these dose-dependent changes in cell index were undetectable using the xCELLigence system (data not shown).

**Figure 3:**
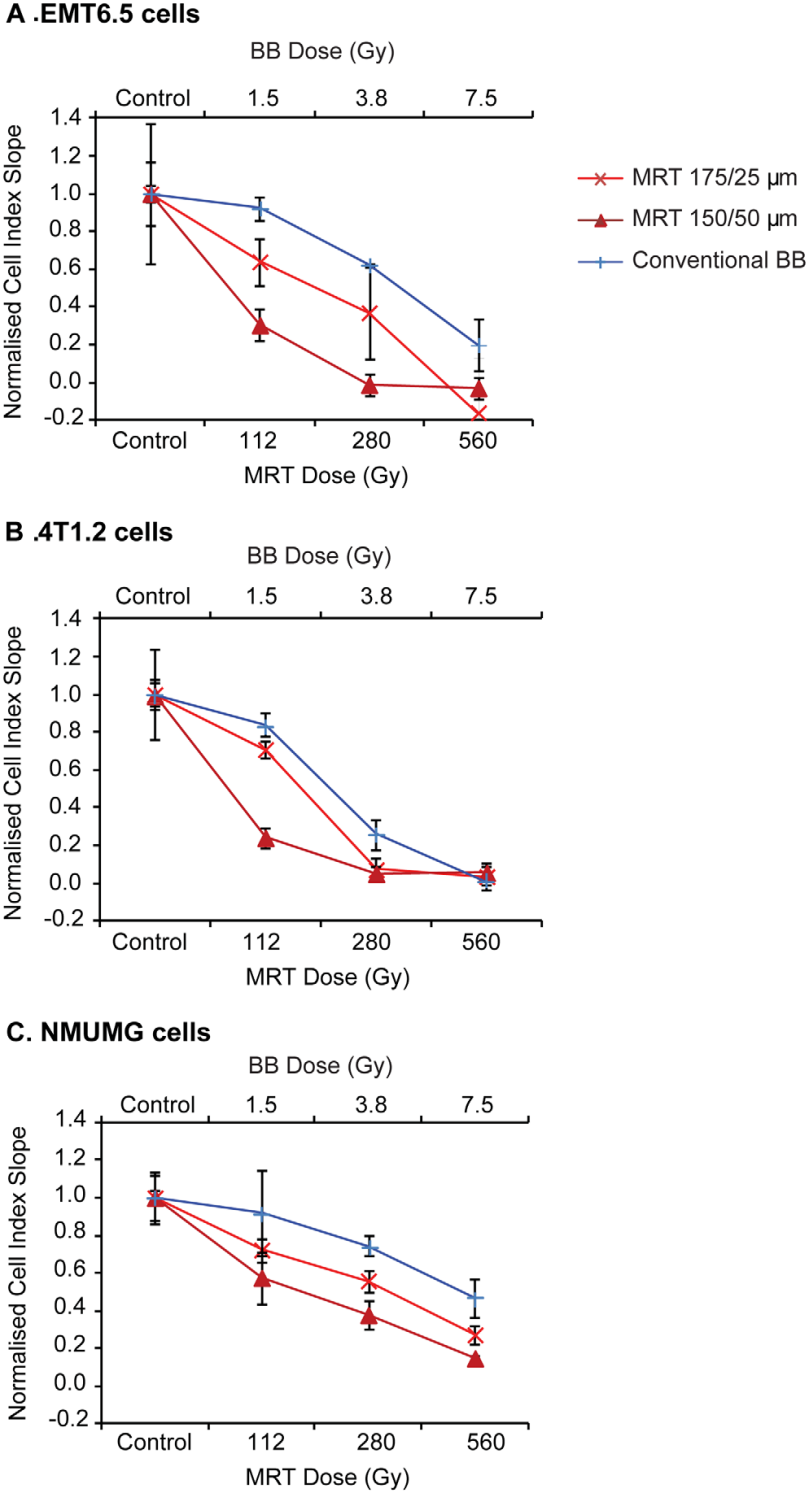
Comparison of cell proliferation response by xCELLigence assays following BB and MRT irradiations. (A) EMT6.5 tumour, (B) 4T1.2 tumour and (C) NMuMG normal mouse mammary epithelial cells following synchrotron BB or MRT 175/25 µm and MRT 150/50 µm irradiation. BB doses were similar to the MRT valley dose determined by Monte Carlo modelling. For all cell lines, the reduction in cell index for 150/50 µm>175/25 µm>BB. Data are represented as mean ± SEM (n = 3).

Figure 3 is a plot of the change in cell index during the logarithmic growth phase, for each cell line with increasing BB/MRT dose. For all three cell lines (Figure 3 A–C), the reduction in cell index was greater for 175/25 µm MRT than BB (BB≤ MRT valley dose). Further, the 150/50 µm collimator reduced cell index compared to the 175/25 µm collimator.

#### Calculating dose equivalence between BB and MRT (clonogenic assays)

Dose response curves of the form 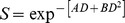 were fitted to the BB data from Figure 2 A–C (R^2^−0.9–1). Using this linear quadratic model, for all cell lines we interpolated BB doses that would generate survival fraction values equivalent to MRT 175/25 µm or MRT 150/50 µm (Table 1). For EMT6.5, we obtained BB dose equivalence data for all MRT 175/25 µm doses and for 4T1.2 and NMuMG for all doses except 560 Gy (out of range). Similarly, BB dose equivalence data were obtained for all cell lines for all MRT 150/50 µm doses except for 560 Gy (out of range). These results are presented in Table 1. The interpolated BB doses for a given MRT dose/configuration were similar for all three cell lines.

**Table 1:** Interpolated equivalent broadbeam doses for increasing MRT doses.

	#Equivalent BB doses (Gy)			
Cell line	50 Gy MRT	112 Gy MRT	280 Gy MRT	560 Gy MRT
*EMT6.5 175/25*	*3.2±0.4*	*5.0±0.2*	*7.7±0.4*	*9.3±0.4*
EMT6.5 150/50	5.5±0.3	8.4±0.7	10.6±0.1	OOR*
4T1.2 175/25	3.1±0.4	4.6±0.5	8.70±0.98	OOR*
4T1.2 150/50	4.2±0.4	6.4±0.7	9.3±0.8	OOR*
NMuMG 175/25	2.9	5.6±0.7	9.6±0.5	OOR*
NMuMG 150/50	4.3	8.0±0.4	OOR8	OOR*

# Linear quadratic dose response curves were fitted to the clonogenic data from Figure 2.

*OOR- out of range.

#### Calculating dose equivalence between BB and MRT (xCELLigence assays)

Sigmoidal dose response curves were generated from the BB data in Figure 3 A–C using xCELLigence RTCA software v1.1 (R^2^−0.8–1). Using this model, BB dose equivalence data were obtained for all cell lines at all doses except 560 Gy (which was only obtained for 4T1.2 and NMuMG; Table 2). The interpolated BB doses for a given MRT dose/configuration were similar for EMT6.5 and NMuMG but not 4T1.2 which showed only a minor increase in BB dose equivalence values with increasing MRT dose.

**Table 2:** Interpolated equivalent broadbeam doses for increasing MRT doses.

	#Equivalent BB doses (Gy)			
Cell line	50 Gy MRT	112 Gy MRT	280 Gy MRT	560 Gy MRT
EMT6.5 175/25	2.8	5.6±0.1	8.2±1.1	OOR*
EMT6.5 150/50	3.7	6.9±0.6	14.8±2.7	OOR*
4T1.2 175/25	1.9	2.3±0.2	3.8±0.2	4.8±0.6
4T1.2 150/50	2.4	2.9±0.4	4.7±0.8	4.5±0.1
NMuMG 175/25	2.0	5.3±0.4	7.7±1.3	12.7
NMuMG 150/50	3.9	6.8±1.4	9.3±0.3	OOR*

# Sigmoidal dose response curves were fitted to the xCELLigence data from Figure 3.

*OOR- out of range.

### Experimental Session (II) at the Australian Synchrotron

#### Testing for dose equivalence between BB and MRT using clonogenic assays

Based on the MRT equivalent BB doses for EMT6.5 cells (175/25 µm) from Table 1 as an example), we compared the following – 3.2 Gy BB vs 50 Gy MRT, 5 Gy BB vs 112 Gy MRT, 7.7 Gy BB vs 280 Gy MRT and 9 Gy BB vs 560 Gy MRT. We repeated the experiments using EMT6.5ch, 4T1ch5 and SaOS-2 cell lines.

**Figure 4:**
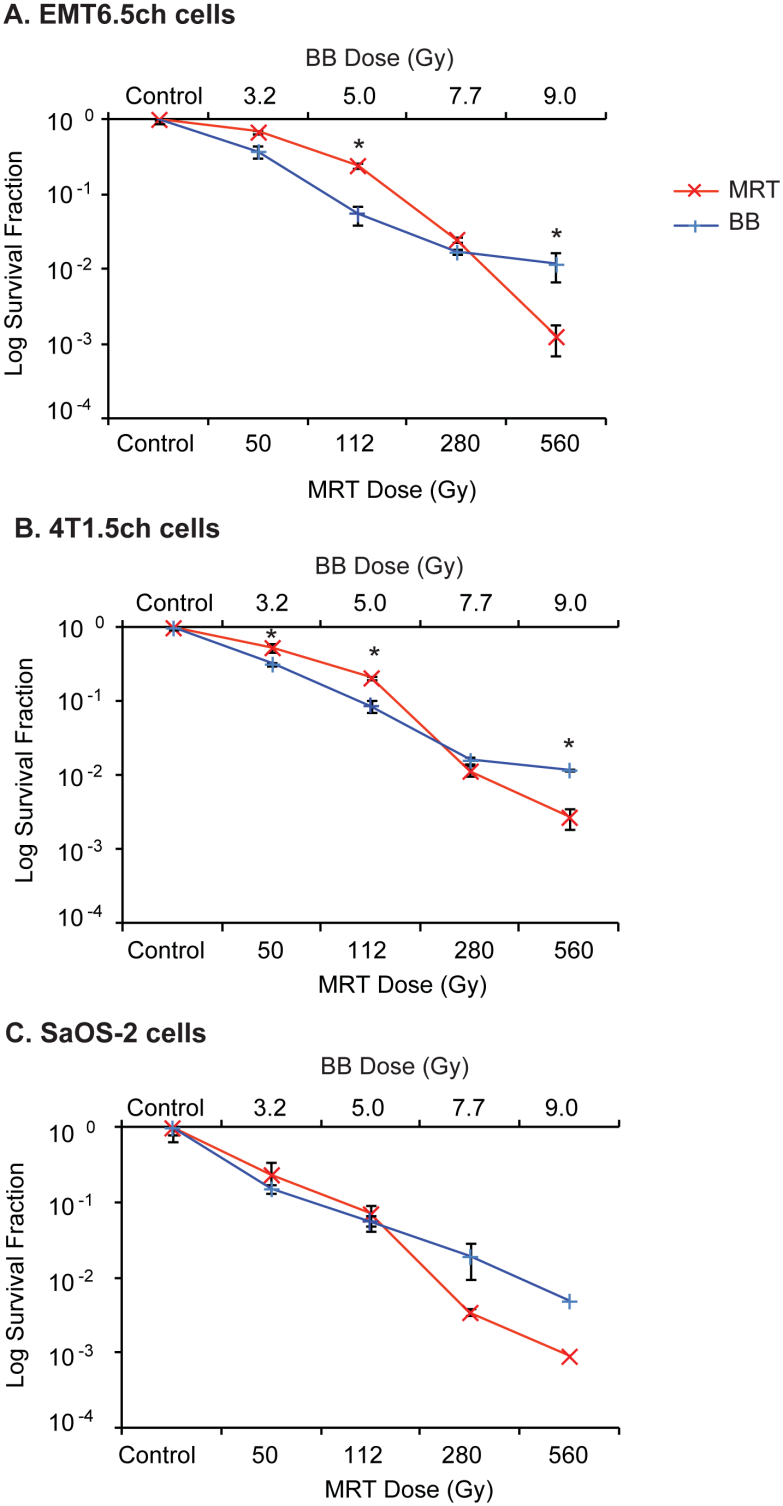
Testing for dose equivalence between BB and MRT by clonogenic assays. Comparison of clonogenic survival of (A) EMT6.5ch tumour, (B) 4T1Ch5 tumour and (C) SaOS-2 tumour cells following MRT 175/25 or synchrotron BB at equivalent doses. Data are represented as mean ± SEM for EMT6.5ch (n = 3), 4T1Ch5 (n = 3) and SaOS-2 (n = 2); *p<0.05. EMT6.5ch – mouse mammary tumour cells, 4T1Ch5– mouse mammary tumour cells, SaOS-2– osteocarcinoma cells.

For EMT6.5ch, clonogenic survival at 3.2 Gy BB and 7.7 Gy BB was not significantly different compared to 50 Gy MRT and 280 Gy MRT respectively (Figure 4 A). However, clonogenic survival at 5 Gy BB was significantly lower compared to 112 Gy MRT (p = 0.003).

For 4T1ch5, clonogenic survival at 7.7 Gy BB was not significantly different to 280 Gy MRT (Figure 4 B). However, 3.2 Gy BB and 5 Gy BB decreased clonogenic survival significantly compared to 50 Gy MRT (p = 0.015) and 112 Gy MRT (p = 0.0008) respectively (Figure 4 B).

For SaOS-2, there were insufficient data points for statistical analysis (n = 2). However, clonogenic survival at 3.2 Gy BB and 5 Gy BB did not appear to be different to 50 Gy MRT and 112 Gy MRT respectively, whereas 280 Gy MRT lowered clonogenic survival compared to 7.7 Gy BB.

For all cell lines, 560 Gy MRT reduced clonogenic survival compared to 9 Gy BB (Figure 4 A–C). This reduction was statistically significant in EMT6.5ch (p = 0.006) and 4T1ch5 (p = 0.03).

#### Testing for dose equivalence between BB and MRT using xCELLigence assays

We compared the following – 3.2 Gy BB vs 50 Gy MRT, 5 Gy BB vs 112 Gy MRT, 7.7 Gy BB vs 280 Gy MRT and 9 Gy BB vs 560 Gy MRT and 10.3 Gy vs 1000 Gy MRT in EMT6.5ch, 4T1ch5 and SaOS-2 cell lines.

**Figure 5:**
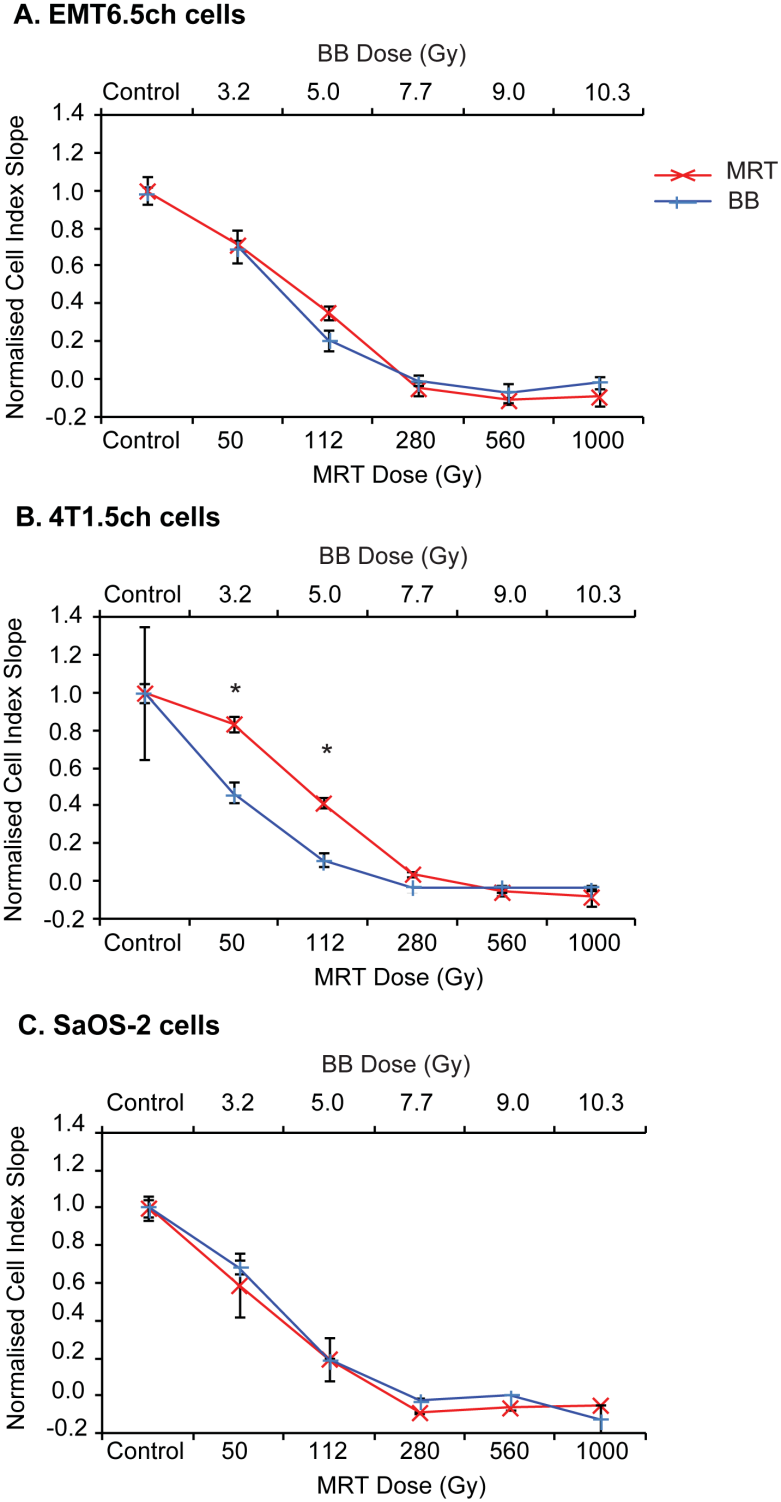
Testing for dose equivalence between BB and MRT by xCELLigence assays. Comparison of cell proliferation response by xCELLigence assays of (A) EMT6.5ch tumour, (B) 4T1Ch5 tumour and (C) SaOS-2 tumour cells following MRT 175/25 or synchrotron BB irradiation at equivalent doses. Data are represented as mean ± SEM (n = 3); *p<0.05. EMT6.5ch–mouse mammary tumour cells, 4T1Ch5–mouse mammary tumour cells, SaOS-2–osteocarcinoma cells.

For EMT6.5ch, the effects of 3.2 Gy, 7.7 Gy, 9 Gy and 10.3 Gy BB on the cell index were not significantly different to 50 Gy, 280 Gy, 560 Gy and 1000 Gy MRT respectively (Figure 5 A). Although 5 Gy BB decreased cell index compared to 112 Gy MRT, this decrease did not reach statistical significance (p = 0.09).

For 4T1ch5, there were no significant differences in the cell index between 7.7 Gy, 9 Gy and 10.3 Gy BB and 280 Gy, 560 Gy and 1000 Gy MRT (Figure 5 B). However, 3.2 Gy BB and 5 Gy BB decreased cell proliferation significantly compared to 50 Gy MRT (p = 0.03) and 112 Gy MRT (p = 0.003) respectively (Figure 5 B). For SaOS-2, there were insufficient data points for statistical analysis (n = 2). There did not appear to be any differences in the rate of cell proliferation of SaOS-2 cells at the matched BB/MRT doses used (Figure 5 C).

#### Calculating dose equivalence between BB and MRT (clonogenic assays)

Dose response curves of the form 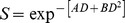 were fitted to the BB data from Figure 4 A-C. Using this model, we interpolated BB doses that would generate survival fraction values equivalent to MRT 175/25 µm (Table 3). BB dose equivalence data were obtained for all cell lines for all MRT 175/25 µm doses. The interpolated BB doses for a given MRT dose were similar for all three cell lines.

**Table 3:** Interpolated equivalent broadbeam doses for increasing MRT doses.

	#Equivalent BB doses (Gy)			
Cell line	50 Gy	112 Gy	280 Gy	560 Gy
EMT6.5ch	1.50±0.28	3.4±0.1	9.70±0.37	12.50±0.43
4T1ch55	0.98±0.06	2.90±0.11	7.1±0.2	12.0±0.7
SaOS-2	2.40±0.69	4.6±0.6	11.5	11

# Dose response curves of the form 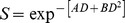 were fitted to the clonogenic data from Figure 4.

#### Calculating dose equivalence between BB and MRT (xCELLigence assays)

Sigmoidal dose response curves were generated from the BB data from Figure 5 A–C using xCELLigence RTCA software v1.1. Using this model, we interpolated BB doses equivalent to MRT 175/25 µm (Table 4). The interpolated BB doses for a given MRT dose were similar for all three cell lines.

**Table 4:** Interpolated equivalent broadbeam doses for increasing MRT doses.

	Equivalent BB doses (Gy)			
Cell line	50 Gy	112 Gy	280 Gy	560 Gy
EMT6.5ch	3.20±0.24	4.40±0.04	8.8±1.4	OOR*
4T1ch5	1.80±0.15	3.20±0.02	5.4±0.5	OOR*
SaOS-2	3.80±0.62	4.8±0.4	7.9±0.8	OOR*

Sigmoidal dose response curves were fitted to the xCELLigence data from Figure 5. BB doses equivalent to MRT 175/25 peak doses were interpolated.

*OOR-out of range.

## Discussion

In this study, we provide the first *in vitro* dose equivalence data for synchrotron MRT and BB radiotherapy using six different cell lines. We determined dose equivalence between MRT and BB using conventional, clonogenic and novel, RT-CIS/xCELLigence *in vitro* assays (Tables 1–4). Our results provide evidence that it is possible to determine dose equivalence between BB and MRT using *in vitro* assays. Further, we have also shown that xCELLigence RT-CIS may be used as a rapid, high-throughput alternative to clonogenic assays for determining dose equivalence between MRT and BB.

The Monte Carlo method predicts a physical deposition of energy. The biologically equivalent dose described here is not the same as the physical valley dose, but rather is the dose of BB radiation which elicits the same biological effect in cells as MRT, be it clonogenic capacity or cell proliferation index. The biological equivalent dose takes into account the different dose components of MRT; peak, valley, integrated dose. This equivalence in dose is the novel aspect of our work; for a given MRT dose distribution, we can state what the equivalent biological response is with a BB dose distribution.

Our group have previously reported on the use of the Gamma-H2AX assay as a biodosimeter to estimate the valley dose in MRT [24]. The G-H2AX-measured dose (a function of foci number) can be compared directly with the Monte Carlo-predicted dose (or some other physically determined dose) since there is an almost linear relationship between the number of DNA double strand breaks (visualised as foci) and the absorbed dose. However, G-H2AX is limited in its ability to determine a biologically equivalent dose other than showing a certain valley dose of MRT causes the same number of DNA double strand breaks as a certain dose of BB radiation.

We began by comparing MRT with BB doses equivalent to the Monte Carlo–calculated MRT valley dose, hypothesizing (as a starting point) that the valley dose was the dominant influence for determining the *in vitro* response to MRT. We then fit our *in vitro* data from clonogenic assays and RT-CIS/xCELLigence assays to two models - the linear-quadratic (LQ) model and the sigmoidal dose-response model respectively. These models have been previously described in the literature [25] [26] to detail the relationship between the radiation dose and the cellular response with respect to these two *in vitro* assays. Based on these models we predicted dose equivalence by data interpolation.

We selected MRT equivalent BB doses relevant to the EMT6.5 tumour cells as an arbitrary starting point and tested these in EMT6.5ch, 4T1ch5 and SaOS-2 cell lines during our second session at the Australian synchrotron. These cell lines were selected because they would be used in future *in vivo* irradiation studies. Importantly, for EMT6.5ch and SaOS-2 cell lines, the BB doses calculated to give equivalence to the matched MRT doses gave similar outcomes (except at 560 Gy). This was not the case for 4T1ch5 with 3.2 Gy BB and 5 Gy BB giving reduced clonogenic survival compared to 50 Gy MRT and 112 Gy MRT. It is important to note that each cell type has a characteristic and repeatable radiation response profile. It is not unreasonable to assume that dose equivalence between BB and MRT may vary from cell line to cell line and determination of dose equivalence by this approach may need to be performed specifically using the cell line in question. What is noteworthy is that for the first time, we have devised an actual approach for determining biological dose equivalence between MRT and BB.

For a given cell line, the values for BB dose equivalence (per MRT configuration) did not vary greatly between clonogenic and RT-CIS/xCELLigence assays (Tables 1 vs 2 and Table 3 vs 4). This similarity between assays (also demonstrated by [13]), was remarkable given that the parameters being measured were completely different. RT-CIS provides data on several parameters that pertain to the biological status of a cell, including cell number, metabolism and morphology. In contrast, clonogenic assays are endpoint assays solely measuring the ability of cells to survive irradiation, proliferate and form a colony of daughter cells over 2–4 weeks. The rapid nature of RT-CIS provides us with a method to conduct high throughput screening of the biological effect of radiation (at a number of doses) on a number of cell lines in a time and cost-saving manner in comparison to traditional clonogenic assays.

The values for BB dose equivalence for a given MRT configuration were similar both within an assay as well as between assays for all cell lines apart from 4T1.2 (Tables 1–4). Dose equivalence could not be accurately determined for 4T1.2 cells by RT-CIS/xCELLigence assays (Table 2). This discrepancy could be because the RT-CIS profiles for this cell line at 280 Gy and 560 Gy reached a plateau at a higher cell index than that at 7.5 Gy BB (Figure S1). It is possible that MRT (and not BB) alters other aspects of cell behaviour such that the resulting cell index is not representative of cell number alone. Further, these effects may occur in a cell specific manner. Of note, MRT may influence the migration of tumour cells [8] and such an event is likely to influence cell index.

RT-CIS/xCELLigence could not be accurately used to verify biological dose equivalence for doses above 7.7 Gy BB or 280 Gy MRT for some cell lines (Figure 5). This is because at such doses the RT-CIS profiles reached a plateau where no change in cell index was observed over time (slope = zero) with increasing radiation dose. Some cells did not even enter their log phase of growth at these doses (e.g. 4T1.2, 4T1.5 and SaOS-2; Figures S1 and S2). This reflects the upper sensitivity limit of this assay and can be partially overcome by directly comparing RT-CIS profiles of cells from BB and MRT experiments on the same xCELLigence E-plate. This was not logistically possible in our current set of experiments and warrants further investigation.

Another important finding arising from this work is that the MRT *valley* dose alone does not determine the acute cellular response to MRT–the biological effect was greater for MRT compared to BB at doses similar to or slightly greater than the predicted MRT valley dose. The study by Priyadarshika et al. [27] concluded that the acute response of normal mouse skin to MRT may be dictated by the integrated MRT dose rather than the peak or valley dose. The integrated doses for the 175/25 and 150/50 µm collimator geometries are approximately a factor of 1/8 and 1/4 the peak dose respectively [28]. Therefore for the peak microbeam doses of 50, 112, 280, and 560 Gy in this current study, the integrated doses were approximately 6, 14, 35, and 70 Gy for the 175/25 µm collimator geometry and 12, 28, 70, and 140 Gy for the 150/50 µm collimator geometry. Therefore, the integrated doses were not_a useful indicator of the biological equivalence of MRT and BB radiation for these *in vitro* experiments since they were larger than our measured/predicted BB doses. A dosimetric discrepancy on the beamline or at the WEHI’s Co^60^ source may be the cause of the significant differences in clonogenic survival observed at a dose of 7.5 Gy.

The radiation-induced bystander effect is another mechanism which may explain the greater than expected cell kill with MRT [29], [30]. Radiation-induced bystander effects have been shown to be involved in studies using modulated radiation beams. The study by Asur et al. 2012 [31] using GRID therapy in mammary carcinoma (SCK) and head and neck cancer (SCCVII) cells showed identical cell survival fraction between medium transfer bystander and direct bystander effect between cells. While Fernandez-Palomo (2013) [32] using synchrotron MRT with doses up to 350 Gy on tumour bearing-mice showed bystander effects in non-irradiated tumour sites. These results showed bystander effects cause cell death to the abscopal area and might explain greater cell killing rate in our study compared to the calculated dose using Monte Carlo simulation.

Our results also showed the importance of the geometrical configuration of the microbeam collimator; with the 150/50 µm collimator geometry reducing clonogenic survival and cell viability compared to the 175/25 µm geometry. An analogous finding was reported in the work of Regnard et al. [6] in which reduced survival in glioma-inoculated rats was observed for a collimator geometry with a 200 µm centre-to-centre spacing compared to a 100 µm spacing geometry.

The dosimetry for the MRT studies presented here was based entirely on Monte Carlo computer simulation. These computer models of X-ray dose deposition do have limitations and approximations (especially in zones of high dose gradient), which ultimately affect the accuracy of the predictions. Certain physical processes such as polarisation and low energy X-ray scatter are perhaps not fully accounted for. The collimator geometry and the overall beamline geometry is also idealised. It is likely the Monte Carlo simulations under-estimates the ‘true’ valley dose, and therefore overestimates the PVDR as a result.

The low energy X-rays used in MRT limits the depth of penetration of the microbeams. For example, the half value layer of the X-ray beam is about 50 mm in water. Deep-seated tumour sites in the pelvis (e.g. prostate cancer) are therefore unlikely to be treatable by MRT. However, MRT may have applications for treating tumours in ‘shallower’ clinical sites, for example; skin, bone (osteosarcoma), breast, tumours in the head & neck and upper thorax regions (e.g. lung cancer). The synchrotron radiotherapy research groups at the ESRF in France have a long-standing research programme into treating primary and metastatic brain tumours with synchrotron radiation.

The results of this study will be informative for MRT dose protocols for future veterinary and clinical trials at the Australian Synchrotron. The long term vision of this work is the safe implementation of MRT as a clinical radiotherapy treatment for cancer. The significance of developing a new radiotherapy approach with an improved therapeutic index is enormous, with the potential for improved survival outcomes for a large percentage of people with cancer.

## Conclusion

In conclusion, we have established a novel method to determine dose equivalence between BB and MRT using two different biological assays. Overall, these studies will improve our understanding of the effectiveness and limitations of MRT, and are essential steps in the pathway towards designing optimal therapeutic approaches and potential combinatorial therapies for MRT. Knowledge of the radiobiological response to MRT at a cellular level is important because the tolerance and repair of cells may inform current radiotherapy paradigms and alter the existing treatment regimens used for tumour control.

## Supporting Information

Figure S1 **xCELLigence profiles of EMT6.5, 4T1.2 and NMuMG cells following BB and MRT irradiations.** (A) EMT6.5 tumour, (B) 4T1.2 tumour and (C) NMuMG normal mouse mammary epithelial cells following conventional BB or MRT 175/25 and MRT 150/50 irradiation. Data shown are mean± SD for each cell line.
(TIF)

Figure S2 **xCELLigence profiles of EMT6.5ch, 4T1ch5 and SaOS-2 tumour cells following BB and MRT irradiations.** (A) EMT6.5ch tumour, (B) 4T1Ch5 tumour and (C) SaOS-2 tumour cells following conventional BB or MRT 175/25 irradiation. Data shown are mean± SD for each cell line.
(TIF)

Table S1 LQ constants and standard error.
(DOCX)

Material S1 **A GEANT4 Monte Carlo simulation of microbeam radiation therapy on the imaging & medical beamline of the Australian Synchrotron.**
(DOCX)
