# Supplementary figures and images for: An Evaluation of Dose Equivalence between Synchrotron Microbeam Radiation Therapy and Conventional Broadbeam Radiation Using Clonogenic and Cell Impedance Assays
Source: PLoS One. 2014 Jun 19;9(6):e100547. doi: 10.1371/journal.pone.0100547 (PMC4063937; doi:10.1371/journal.pone.0100547)

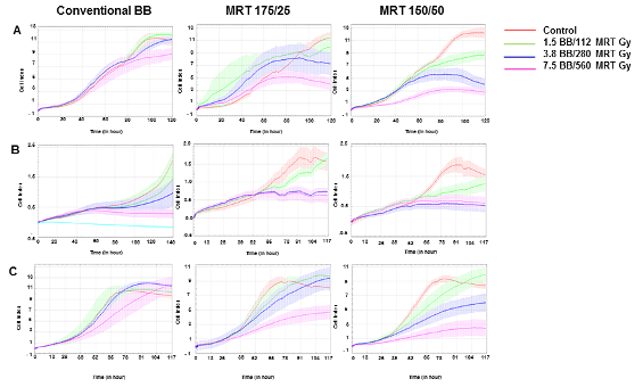

Supplement: Figure S1 — xCELLigence profiles of EMT6.5, 4T1.2 and NMuMG cells following BB and MRT irradiations. (A) EMT6.5 tumour, (B) 4T1.2 tumour and (C) NMuMG normal mouse mammary epithelial cells following conventional BB or MRT 175/25 and MRT 150/50 irradiation. Data shown are mean± SD for each cell line. (TIF) [file pone.0100547.s001.tif]

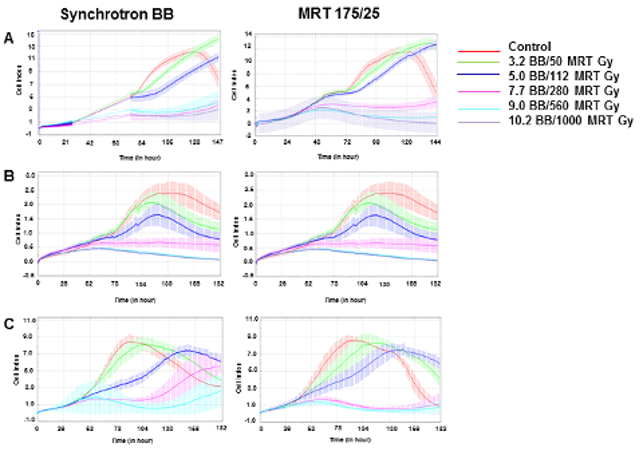

Supplement: Figure S2 — xCELLigence profiles of EMT6.5ch, 4T1ch5 and SaOS-2 tumour cells following BB and MRT irradiations. (A) EMT6.5ch tumour, (B) 4T1Ch5 tumour and (C) SaOS-2 tumour cells following conventional BB or MRT 175/25 irradiation. Data shown are mean± SD for each cell line. (TIF) [file pone.0100547.s002.tif]
