# Supplementary material for: An Evaluation of Dose Equivalence between Synchrotron Microbeam Radiation Therapy and Conventional Broadbeam Radiation Using Clonogenic and Cell Impedance Assays
Source: PLoS One. 2014 Jun 19;9(6):e100547. doi: 10.1371/journal.pone.0100547 (PMC4063937; doi:10.1371/journal.pone.0100547)
Supplement: Table S1 — LQ constants and standard error. (DOCX) [file pone.0100547.s003.docx]

Supplementary Table 1: LQ constants and standard error

***1.0 Experimental session (I) at the Australian Synchrotron***

1.1 Clonogenic assays

| Mean | EMT6.5 | 4T1.2 | NMUMG |
| --- | --- | --- | --- |
| B_0_ | -0.0028 | -0.0629 | -0.0352 |
| B_1_ | -0.1346 | -0.0088 | -0.0734 |
| B_2_ | -0.0036 | -0.0197 | -0.0083 |
|  |  |  |  |
| Std. Error | 0.0251 | 0.0989 | 0.0464 |
| B_0_ | 0.0181 | 0.0711 | 0.0178 |
| B_1_ | 0.0023 | 0.0089 | 0.0011 |
| B_2_ | -0.0028 | -0.0629 | -0.0352 |

1.2 xCELLigence cell index slope

| Mean | EMT6.5 | 4T1.2 | NMUMG |
| --- | --- | --- | --- |
| Bottom | 0.1915 | 0.0306 | -0.1973 |
| Top | 1.0350 | 1.0220 | 1.0610 |
| LogIC50 | 0.9462 | 0.5204 | 1.1410 |
| IC50 | 8.8360 | 3.3140 | 13.8400 |
| Span | 0.8433 | 0.9916 | 1.2590 |
|  |  |  |  |
| Std. Error |  |  |  |
| Bottom | 0.0832 | 0.1045 | 0.2564 |
| Top | 0.0447 | 0.0665 | 0.0819 |
| LogIC50 | 0.1418 | 0.1956 | 0.2375 |
| IC50 | 0.0831 | 0.1139 | 0.2395 |
| Span | 0.1915 | 0.0306 | -0.1973 |

***2.0 Experimental session (I) at the Australian Synchrotron***

2.1 Clonogenic assays

| Mean | EMT6.5ch | 4T1.5ch | SaOS-2 |
| --- | --- | --- | --- |
| B_0_ | 0.0486 | 0.0494 | -0.0467 |
| B_1_ | -0.2218 | -0.2216 | -0.2564 |
| B_2_ | 0.0016 | -0.0016 | 0.0037 |
|  |  |  |  |
| Std. Error |  |  |  |
| B_0_ | 0.0931 | 0.1408 | 0.1086 |
| B_1_ | 0.0467 | 0.0706 | 0.0545 |
| B_2_ | 0.0049 | 0.0074 | 0.0056 |

2.2 xCELLigence cell index slope

| Mean | EMT6.5ch | 4T1.5ch | SaOS-2 |
| --- | --- | --- | --- |
| Bottom | 0.1999 | 0.1143 | 0.1222 |
| Top | 0.9762 | 0.9990 | 1.0010 |
| LogIC50 | 0.6966 | 0.5308 | 0.6516 |
| HillSlope | -3.7600 | -2.8810 | -4.1280 |
| IC50 | 4.9730 | 3.3950 | 4.4830 |
| Span | 0.7763 | 0.8847 | 0.8788 |
|  |  |  |  |
| Std. Error |  |  |  |
| Bottom |  |  |  |
| Top | 0.0382 | 0.0446 | 0.0355 |
| LogIC50 | 0.0422 | 0.0525 | 0.0380 |
| IC50 | 0.0303 | 0.0432 | 0.0252 |
| Span | 0.7644 | 0.8754 | 0.8294 |

***3.0 Example of the LQ fit***
