# Supplementary material for: An Evaluation of Dose Equivalence between Synchrotron Microbeam Radiation Therapy and Conventional Broadbeam Radiation Using Clonogenic and Cell Impedance Assays
Source: PLoS One. 2014 Jun 19;9(6):e100547. doi: 10.1371/journal.pone.0100547 (PMC4063937; doi:10.1371/journal.pone.0100547)
Supplement: Material S1 — A GEANT4 Monte Carlo simulation of microbeam radiation therapy on the imaging & medical beamline of the Australian Synchrotron. (DOCX) [file pone.0100547.s004.docx]

**SUPPLEMENTARY MATERIAL 1**

A GEANT4 Monte Carlo simulation of microbeam radiation therapy on the imaging & medical beamline of the Australian Synchrotron

Jeffrey C. Crosbie^1,2^, Jeremy Brown^3^, Matthew Dimmock^4^, Gareth Skinner, Iwan Cornelius^5,6^, Toby Beveridge, & Stewart M. Midgley^3^

^1^University of Melbourne Department of Obstetrics & Gynaecology, The Royal Women’s Hospital, Parkville, VIC 3052,

^2^William Buckland Radiotherapy Centre, Alfred Hospital, Melbourne, VIC 3004,

^3^School of Physics and ^4^Department of Medical Imaging & Radiation Sciences, Monash University, Clayton, VIC 3800,

^5^Centre for Medical Radiation Physics, University of Wollongong, NSW 2522

^6^Imaging & Medical Beamline, The Australian Synchrotron, Clayton, 3168

**OVERVIEW**

GEANT4 (v 4.9.2); a Monte Carlo radiation transport modelling toolkit [1], was used to construct a model of the microbeam radiotherapy measurements undertaken at the IMBL. The model simulated the transport of the X-rays emitted from a multi-pole wiggler, through the beam defining slits (50 mm thick copper blocks), the tungsten carbide/kapton collimator and into a radiochromic film detector 2 mm deep in a water phantom, which was positioned 20.55 m from the wiggler exit. The source size of the wiggler was modelled as a two dimensional Gaussian field with the spatial dimensions 754 µm horizontally and 38 µm vertically at full width half maximum, and a divergence of 5 mrad and 0.3 mrad respectively [2]. High precision definition of the source allows us to readily account for the beam divergence which influences the valley dose as reported by Nettelbeck et al. [3]. The vertical opening of the slits was on the order of 0.8 mm for each simulation, as it was for the actual irradiations. The horizontal opening of the slits varied depending on the simulation/irradiation conditions.

The tungsten carbide/kapton collimator was modelled as a stack of alternating sheets, sandwiched between two exterior sheets of tungsten carbide, with density provided by the National Institute of Standards and Technology (NIST) database [4]. The energy of the simulated photons were randomly sampled from a histogram of the IMBL's energy spectrum, which was determined using the SPECTRA program of Tanaka and Kitamura [5]. The low energy electromagnetic and atomic de-excitation physics packages of Geant4 with a cut-off step length of 0.001 mm were used to account for the dominant interactions of kilovoltage X-rays with matter.

Batches of twenty simulations of 1 x 10^9^ histories were simulated for each collimator geometry using the ‘Trifid’ high performance computing cluster provided by the Victorian Partnership for Advanced Computing (VPAC). Each collimator geometry took approximately 3 days to simulate using the Trifid cluster. The raw data were histogrammed using an in-house designed program and plotted to give a final profile. The step-and-shoot nature of the irradiations was simulated by summing sets of 5 microbeams together at intervals of 1 mm, to create arbitrarily long field size dimensions.

We anticipate a more in-depth description of the Monte Carlo model, as well as detailed results, in a future publication dedicated to the dosimetric aspects of MRT research on the IMBL.

[1] Agostinelli S, Allison J, Amako K, et al 2003 G4--a simulation toolkit *Nucl. Instruments Methods Phys. Res. Sect. A Accel. Spectrometers, Detect. Assoc. Equip.* **506** 250–303

[2] Stevenson A W, Mayo S C, Häusermann D, et al 2010 First experiments on the Australian Synchrotron Imaging and Medical beamline, including investigations of the effective source size in respect of X-ray imaging. *J. Synchrotron Radiat.* **17** 75–80

[3] Nettelbeck H, Takacs G J, Lerch M L and Rosenfeld A B 2009 Microbeam radiation therapy: a Monte Carlo study of the influence of the source, multislit collimator, and beam divergence on microbeams *Med Phys* **36** 447–56

[4] Berger M J, Hubbell J H, Seltzer S M, et al 1998 XCOM: Photon Cross Sections Database

[5] Tanaka T and Kitamura H 2001 SPECTRA: a synchrotron radiation calculation code *J. Synchrotron Radiat.* **8** 1221–8
